# Prevalence of dyslipidemia and its associated factors among university academic staff and students in Bangladesh

**DOI:** 10.1186/s12872-023-03399-1

**Published:** 2023-07-21

**Authors:** Nurshad Ali, Rahanuma Raihanu Kathak, Khandaker Atkia Fariha, Abu Taher, Farjana Islam

**Affiliations:** Department of Biochemistry and Molecular Biology, Shahjalal University of Science and Technology, Sylhet, 3114 Bangladesh

**Keywords:** Dyslipidemia, University staff and students, Prevalence, Risk factors, Bangladesh

## Abstract

**Background:**

Dyslipidemia is one of the important contributors to cardiovascular disease and type 2 diabetes. There is little or no information on dyslipidemia among academic staff and students in Bangladesh. Therefore, this study aimed to investigate the prevalence and factors related to dyslipidemia among university academic staff and students in Bangladesh.

**Methods:**

A total of 533 participants (302 academic staff and 231 students) were enrolled in this cross-sectional study. A simple random sampling technique was used to enrol the participants. Fasting blood samples were obtained from the participants, and serum levels of triglycerides (TG), total cholesterol (TC), high-density lipoprotein cholesterol (HDL-C) and low-density lipoprotein cholesterol (LDL-C) were measured using the standard methods. Dyslipidemia was defined according to the National Cholesterol Education Program Adult Treatment Panel III (NCEP-ATP-III) model guideline. Multivariable logistic regression was conducted to identify the factors related to lipid marker abnormalities.

**Results:**

Overall, the prevalence of dyslipidemia was 81.5%, of which 85% was in staff and 76.5% in students. A significant difference was found in the prevalence of dyslipidemia between males and females only in the student group (p < 0.01). Among staff, hypertriglyceridemia prevalence was 49.7%, hypercholesterolemia 23%, high LDL-C 24.7% and low HDL-C 77.3%. On the other hand, hypertriglyceridemia prevalence was 39%, hypercholesterolemia 25.6%, high LDL-C 26.5% and low HDL-C 69.3% among students. The most common lipid abnormality was low HDL-C in both groups. The prevalence of mixed dyslipidemia was 14.2% and 14.1% in staff and students, respectively. According to the regression analysis, increased age, obesity, diabetes, and inadequate physical activity were significantly associated with dyslipidemia.

**Conclusions:**

Dyslipidemia was prevalent among the majority of the study participants. Increased age, obesity, diabetes, and inadequate physical activity were significantly associated with dyslipidemia. The study’s results highlight the importance of implementing interventions to address the associated risk factors of dyslipidemia among academic staff and students in Bangladesh.

## Background

Dyslipidemia is a state that occurs due to the abnormalities of lipids in the blood, such as elevated total cholesterol (TC), elevated triglycerides (TG), low level of high-density lipoprotein cholesterol (HDL-C) and elevated low-density lipoprotein cholesterol (LDL-C). These abnormalities can occur either single or combinedly [1]. Dyslipidemia, especially high levels of LDL-C, is a significant risk factor for cardiovascular disease (CVD), but other forms, such as hypertriglyceridemia, are related to acute pancreatitis and non-alcoholic fatty liver disease [2]. Hypercholesterolemia is the most prevalent form of dyslipidemia and is associated with an increased risk of CVD, with higher levels of LDL-C being the 8th leading risk factor for global death in 2019 [2].

The prevalence of dyslipidemia has increased over the last 3 decades and is considered a health burden globally [2]. CVD is a major cause of global death with a significant number of people dying every year from it than from any other reason [3]. A recent literature review indicated a high prevalence of CVD in the Bangladeshi adult population [4].

The nature of work and working environment may be linked with dyslipidemia. There are many aspects of work that involve less physical activity, unhealthy diets, and physical and mental stress. Generally, employed workers spend a significant portion of their lives at work, and the demands and pressure of the work may affect their food habits, lifestyle and daily activity patterns, which may affect their overall health. A study by Catalina-Romero et al. [5] showed a relationship of job stress with dyslipidemia, even after adjusting for multiple covariates. In another study, job-related mental stress was found to be related to increased levels of blood cholesterol and triglycerides among company workers [6]. Similarly, work at academic institutions is not out of mental and physical stress besides being a significant time of sedentary work; hence academic staff may be at risk of dyslipidemia and related CVD. Sedentary behaviours affect the metabolic profiles that are frequently seen in dyslipidemia [7].

A higher prevalence of dyslipidemia has also been reported among young adults in different countries [8–10]. An increased prevalence of dyslipidemia in young adulthood is a concern as it increases the risk of coronary heart disease in later life [11]. It has been suggested that about 50% of young adults with elevated total cholesterol have five times the risk of coronary heart disease and nine times risk of myocardial infarction than those having low total cholesterol levels over 30 to 40 years of age [12, 13]. As both academic staff and young students are very important groups of the national population. Therefore, determining dyslipidemia prevalence and its related risk factors in these special population groups will be an important step in increasing awareness and prevention of dyslipidemia and related health effects. Therefore, this study was conducted to measure dyslipidemia prevalence and associated factors among university students and academic staff in Bangladesh.

## Methods

### Participant recruitment and study design

This study was a cross-sectional design conducted between February 2019 and January 2020. A total of 533 participants (302 academic staff and 231 students) were recruited from the two universities located in Sylhet and Dhaka districts. All the analyses were conducted at the Biochemistry and Molecular Biology Department of SUST, Sylhet, Bangladesh. Inclusion criteria: (i) willingness to participate; (ii) both sexes and (iii) ≥ 18 years of age. Exclusion criteria: (i) participants with physical dysfunction (ii) participants with infectious disease and liver and kidney diseases (iii) women with pregnancy and nursing mothers and (iv) subjects with incomplete questionnaires or missing blood samples. PASS version 15.0 was used for sample size calculation. A sample size of 480 (270 males and 210 females) was needed to achieve 90% statistical power. A simple random sampling technique was used to enrol the participants. The Ethics Review Committee at the BMB Department, School of Life Sciences, SUST approved this study protocol (ID 02/BMB/2019). Written informed consent was obtained from all study subjects before study commencement. All methods of the study were carried out in accordance with institutional guidelines and regulations.

### Data collection

We used a structured questionnaire for collecting the anthropometric, demographic and lifestyle information described elsewhere [14–22]. Individuals’ body height, weight, and waist and hip circumference (WC and HC, respectively) were measured by trained personnel who were experienced in health-related research. Body mass index (BMI) was determined as the person’s weight in kilograms divided by the square of height in meters. Before blood pressure measurements, the participants were asked to rest for 10 min, and then three consecutive blood pressure measurements were taken 5 min apart. The average of 2nd and 3rd measurements were taken for systolic and diastolic blood pressures (SBP and DBP, respectively). An automated blood pressure measuring device was used for blood pressure measurement (Omron M10, Omron Corporation, Tokyo, Japan).

### Specimen collection and lipid markers measurements

After overnight fasting, venous blood samples were collected from the study subjects in the morning. After centrifugation, serum was isolated and stored at -20^o^C until biochemical analysis. A semi-automatic analyzer (Humalyzer 3000, USA) was used to measure biochemical parameters. Serum levels of TG, TC, HDL-C, LDL-C, and fasting blood glucose were measured using enzymatic colourimetric techniques [23–26].

### Diagnostic criteria

Dyslipidemia was defined according to the NCEP-ATP-III model guideline [27]. Dyslipidemia was defined as having one or more of the following: TC ≥ 200 mg/dL; TG: ≥ 150 mg/dL; LDL-C ≥ 130 mg/dL and HDL-C < 40 mg/dL. Mixed hyperlipidemia was defined as TC ≥ 150 mg/dL plus TG ≥ 200 mg/dL. Isolated dyslipidemia was defined as isolated hypercholesterolemia - a combination of high TC (≥ 200 mg/dL) and normal/low TG (< 150 mg/dL); isolated hypertriglyceridemia - a combination of high TG (> 150 mg/dL) and normal/low TC (< 200 mg/dL); isolated low HDL-C was defined as a combination of low HDL-C (≤ 40 mg/dL) with normal TG and TC. Hypertension was defined as SBP above or equal to 140 mmHg and DBP above or equal to 90 mmHg or self-reported use of antihypertensive medications [28, 29]. Participants with diabetes were identified by checking prescriptions provided by physicians and/or self-reported use of anti-diabetic medications. BMI was divided into normal (18.5–23.0 kg/m^2^), overweight (23.1–27.5 kg/m^2^), and obesity (≥ 27.5 kg/m^2^) [30, 31]. Healthy individuals were defined as both non-hypertensive and non-diabetic. Physical activity was grouped as inadequate (comfortable office work and housework), medium (walking, swimming) and adequate (carrying, lifting, jogging, and/or sports). Smoking was classified as never smokers and current smokers.

### Statistical analyses

Data analyses were performed using SPSS Version 25.0 (IBM, Chicago, IL, USA). Data were presented as mean, frequencies and percentages. Independent sample t-test was used to compare the mean of two given samples and the chi-square test was used to compare categorical variables. Multivariable logistic regression was performed to determine the factors independently associated with lipid marker abnormalities. In regression models, elevated lipid profiles were dependent variables and anthro-demographics and behavioural factors were considered the independent variables. All p-values were two-sided and a p-value < 0.05 was considered statistically significant.

## Results

### Characteristics of the study participants

Table 1 shows the general characteristics of the participants. Among 533 participants, 354 were males and 179 were females. The mean age of the staff and students was 40.5 ± 10.0 years and 21.8 ± 2.0 years, respectively, and there was a significant difference between genders (p < 0.001). Among staff, the mean of BMI, WC, SBP and DBP were higher in males than in females (p < 0.01 at least for all cases). Among students, only WC and DBP showed a significant difference between the gender groups (p < 0.05 at least for both cases). Based on blood pressure and blood glucose concentrations, 34.7% and 14.2% of the academic staff were hypertensive and diabetic, respectively; whereas, 7.9% and 2.6% of the students were hypertensive and diabetic, respectively. Regarding biochemical parameters, the mean level of TC and LDL were higher in male staff; whereas mean TG and HDL were slightly higher in female staff but the differences were not statistically significant between the genders. On the other hand, the mean TG level was significantly higher in male students; whereas the mean HDL level was higher in female students (p < 0.001). About 79% of the academic staff and 81.2% of the students were used to either medium or adequate physical activity. About 11% of the academic staff and 16% of the students were used to smoking.

**Table 1:** Baseline characteristics of the study participants

Variables	Academic staff				Students			
	Total	Male	Female	P-value	Total	Male	Female	P-value
N	302	216	86		231	138	93	
Age (years)	40.5 ± 10.0	41.8 ± 10.0	37.3 ± 8.0	0.000	21.8 ± 2.0	22.2 ± 2.0	21.2 ± 2.0	0.000
Weight (kg)	68.4 ± 9.4	70.2 ± 8.5	63.7 ± 10.0	0.000	59.5 ± 11.9	64.4 ± 11.0	52.2 ± 9.2	0.000
Height (cm)	162.9 ± 8.2	166.6 ± 5.5	153.8 ± 6.3	0.000	162.3 ± 10.9	167.4 ± 6.4	154.7 ± 11.8	0.000
BMI (kg/m^2^)	25.7 ± 3.12	25.26 ± 2.6	26.8 ± 3.8	0.000	22.6 ± 4.4	22.9 ± 3.5	22.1 ± 5.6	0.214
WC (cm)	86.6 ± 8.1	88.3 ± 6.7	84.4 ± 9.4	0.005	79.9 ± 8.6	80.8 ± 8.7	77.7 ± 8.1	0.047
HC (cm)	94.9 ± 7.8	94.0 ± 5.6	96.1 ± 9.9	0.117	92.3 ± 7.9	92.1 ± 7.8	92.6 ± 8.1	0.766
SBP (mmHg)	120.9 ± 13.4	123.3 ± 13.1	115.2 ± 12.4	0.000	120.4 ± 66.8	121.1 ± 11.9	119.2 ± 104.9	0.855
DBP (mmHg)	82.1 ± 10.2	83.9 ± 9.6	77.7 ± 10.4	0.000	74.0 ± 9.2	76.3 ± 8.5	70.5 ± 9.1	0.000
Glucose (mg/dL)	100.7 ± 41.4	99.2 ± 34.2	104.4 ± 54.0	0.292	78.1 ± 19.8	79.2 ± 21.6	75.6 ± 19.8	0.105
TG (mg/dL)	164.4 ± 81.2	159.4 ± 83.6	176.5 ± 74.0	0.119	146.6 ± 94.9	166.2 ± 102.3	114.1 ± 70.7	0.000
TC (mg/dL)	167.4 ± 50.2	169.6 ± 51.1	161.9 ± 47.7	0.204	161.3 ± 52.5	166.3 ± 54.2	152.8 ± 48.9	0.069
LDL (mg/dL)	101.3 ± 48.4	105.2 ± 48.4	91.6 ± 47.3	0.027	100.2 ± 46.3	104.7 ± 47.4	92.8 ± 43.7	0.072
HDL (mg/dL)	34.2 ± 10.8	34.0 ± 10.4	34.6 ± 12.0	0.685	35.9 ± 15.0	33.4 ± 13.42	40.1 ± 16.6	0.003
Hypertensive (%)	104 (34.7)	84 (39.3)	20 (23.3)	0.008	18 (7.9)	14 (10.3)	4 (4.4)	0.107
Diabetic (%)	43 (14.2)	31 (14.4)	12 (14.0)	0.929	6 (2.6)	3 (2.2)	3 (3.2)	0.662
Physical activity (%)								
Inadequate	62 (20.6)	42 (19.3)	20 (23.2)	0.482	44 (18.8)	18 (13.0)	26 (27.6)	0.126
Medium/ Adequate	240 (79.4)	174 (80.7)	66 (76.8)		187 (81.2)	120 (87.0)	67 (72.4)	
Smoking status (%)								
No	268 (89.0)	182 (84.7)	86 (100)	0.000	194 (83.8)	106 (77.4)	93 (100.0)	0.000
Yes	34 (11.0)	34 (15.3)	0 (0)		37 (16.2)	37 (22.6)	0 (0.0)	

Data are presented as mean ± SD or %. P-values are obtained from independent sample t-test for continuous variables and Chi-square test for categorical variables. TG: Triglyceride; TC: Total cholesterol; LDL: Low density lipoprotein; HDL: High density lipoprotein

### Dyslipidemia among study participants

**Fig. 1:**
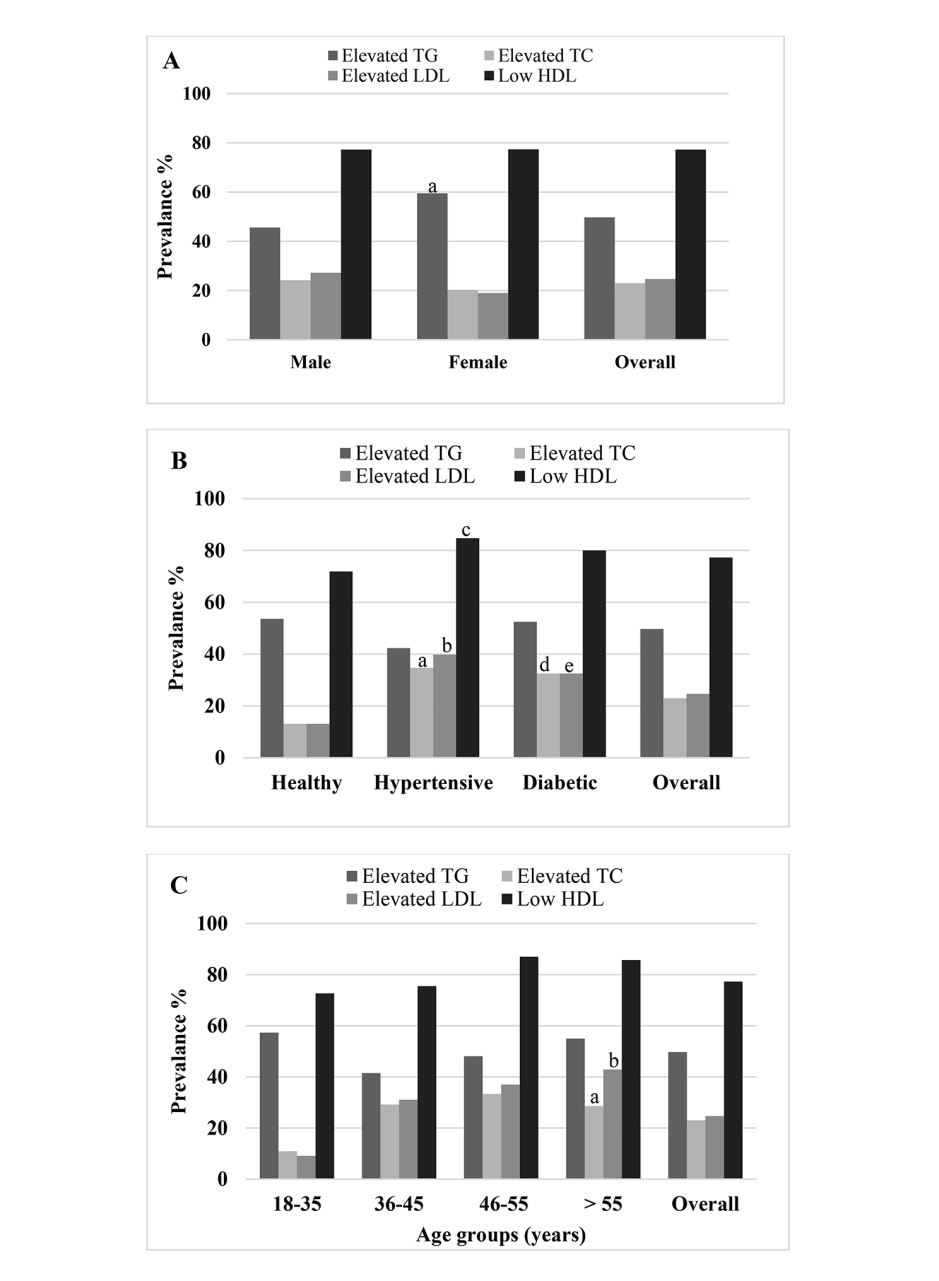
Dyslipidemia prevalence in the sex **(A)**, health status **(B)** and age **(C)** groups among academic staff. In Fig. 1**(A)**, ^a^p<0.05 when the male group is compared to the female group. In Fig. 1**(B)**, ^a,b^p<0.001; ^c^p<0.05 and ^d,e^p<0.01 when the healthy group is compared to hypertensive and diabetic groups respectively. In Fig. 1**(C)**, ^a^p<0.01; ^b^p<0.001 when the lower age groups are compared to the highest age group. P-values are obtained from the chi-square test

**Fig. 2:**
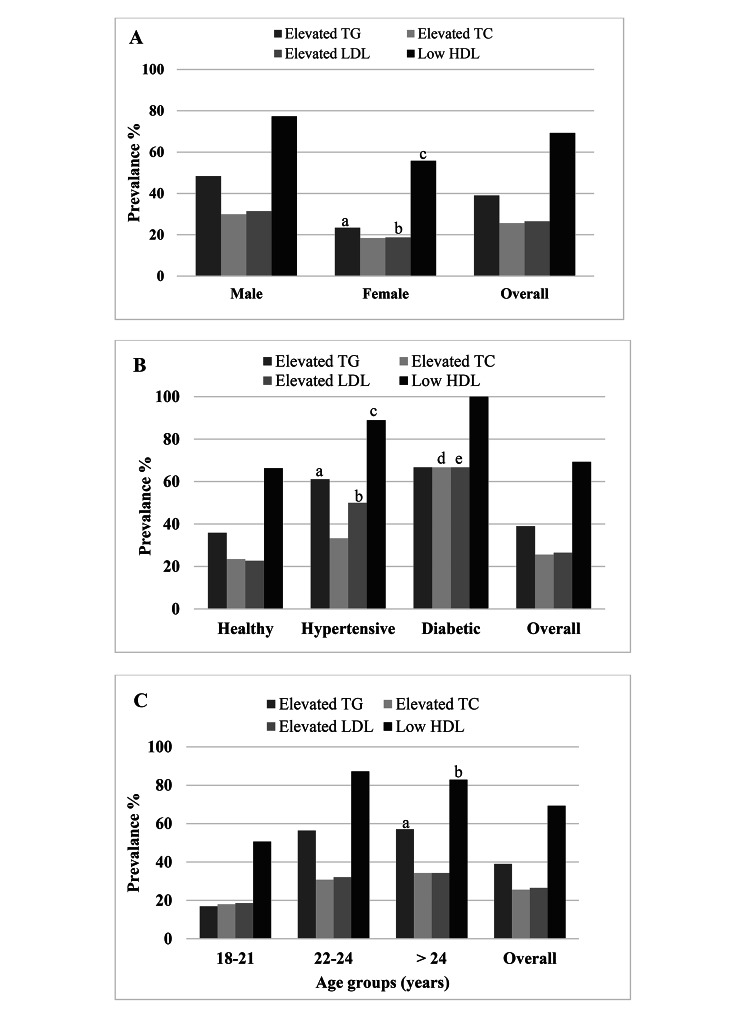
Dyslipidemia prevalence in the sex **(A)**, health status **(B)** and age **(C)** groups among students. In Fig. 2**(A)**, ^a,c^p<0.001; ^b^p<0.05 when the male group is compared to the female group. In Fig. 2**(B)**, ^a,b,c^p<0.05 and ^d,e^p<0.05 when the healthy group is compared to the hypertensive and diabetic groups respectively. In Fig. 2**(C)**, ^a,b^p<0.001 when the lower age groups are compared to the highest age group. P-values are obtained from the chi-square test

Overall, the prevalence of dyslipidemia was 81.5% of which 85% was in academic staff and 76.5% in students (Table 2). No significant difference was found for dyslipidemia prevalence between genders in academic staff (83.5% vs. 88.7%). Whereas, males had a higher prevalence of dyslipidemia than females among the students (82.9% vs. 65.8%, p < 0.01). The lipid levels and prevalence of lipids abnormalities were higher in diabetic and hypertensive individuals than in the control healthy individuals. Among staff, the prevalence of hypertriglyceridemia, hypercholesterolemia, high LDL and low HDL was 49.7%, 23%, 24.7% and 77.3%, respectively (Table 3). On the other hand, this prevalence was 39%, 25.6%, 26.5% and 69.3%, respectively among the students. Among staff, isolated hypertriglyceridemia was 35.5%, isolated hypercholesterolemia was 8.9%, and isolated low HDL-C was 29.6% (Table 4). Mixed hyperlipidemia was prevalent among 14.2% of the staff. In the student’s group, isolated hypertriglyceridemia was 24.9%, isolated hypercholesterolemia 11.3%, and isolated low HDL-C 29.9% (Table 4). Mixed hyperlipidemia was prevalent among 14.1% of the students. Low HDL levels were the main prevalent dyslipidemia in both staff members and students. Among staff, an increasing trend of dyslipidemia was observed in the > 35 years age group and the highest trend was found in the 46–55 years and > 55 years age groups (Fig. 1). In contrast, an increasing trend of dyslipidemia was observed in the > 21 years age group and the highest trend was found in the 22–24 years and > 24 years age groups among the students (Fig. 2).

**Table 2:** Dyslipidemia in different groups

	N	Gender		Dyslipidemia, n (%)			
		Male	Female	Total	Male	Female	P-value
Academic staff	302	216	86	85.0	83.5	88.7	0.217
Students	231	138	93	76.5^a^	82.9	65.8^b^	0.003
Total	533	354	179	81.5	83.3	77.7	0.112

P-values are derived from the chi-square test. ^a^P < 0.01 and ^b^P < 0.001 when the prevalence of dyslipidemia in the staff group is compared to the student group

**Table 3:** Prevalence and levels of lipid markers in different groups

Variables	Overall	Healthy	Hypertensive	Diabetic	^a^P-value	^b^P-value
Academic staff, n	302	155	104	43	-	-
TG (mg/dL)	164.4 ± 81.2	169.4 ± 81.5	153.5 ± 78.2	171.7 ± 86.1	0.125	0.879
Elevated TG, (%)	49.7	53.6	42.3	52.5	0.081	0.902
TC (mg/dL)	167.4 ± 50.2	152.7 ± 47.1	185.1 ± 47.5	180.0 ± 51.5	0.000	0.004
Elevated TC, n (%)	23.0	13.1	34.7	32.5	0.000	0.004
LDL (mg/dL)	101.3 ± 48.4	84.7 ± 43.7	123.6 ± 44.3	110.1 ± 51.6	0.000	0.006
Elevated LDL, n (%)	24.7	13.1	39.8	32.5	0.000	0.004
HDL (mg/dL)	34.2 ± 10.8	35.4 ± 11.6	32.4 ± 8.2	33.8 ± 12.9	0.029	0.493
Low HDL, n (%)	77.3	71.9	84.7	80.0	0.019	0.301
Students, n	231	207	18	6	-	-
TG (mg/dL)	146.6 ± 94.9	141.9 ± 96.3	183.2 ± 81.8	178.5 ± 64.9	0.057	0.233
Elevated TG, n (%)	39.0	35.9	61.1	66.7	0.036	0.125
TC (mg/dL)	161.3 ± 52.5	158.0 ± 53.7	179.9 ± 34.6	203.0 ± 32.9	0.023	0.019
Elevated TC, n (%)	25.6	23.5	33.3	66.7	0.325	0.016
LDL (mg/dL)	100.2 ± 46.3	96.5 ± 46.9	121.8 ± 31.2	143.4 ± 25.5	0.005	0.005
Elevated LDL, n (%)	26.5	22.7	50.0	66.7	0.011	0.013
HDL (mg/dL)	35.9 ± 15.0	36.9 ± 15.6	27.4 ± 6.3	30.6 ± 2.2	0.11	0.000
Low HDL, n (%)	69.3	66.3	88.9	100.0	0.049	0.083

Healthy: both non-hypertensive and non-diabetic. Data are presented as mean ± SD or n (%). ^a^P-value is the difference between the healthy and hypertensive group and ^b^P-value is the difference between the healthy and diabetic group. P-values for mean concentrations are derived from independent sample t-test and P-values for prevalence (%) are obtained from the chi-square test. Elevated TG: TG ≥ 150 mg/dL; Elevated TC: TC ≥ 200 mg/dL; Elevated LDL: LDL ≥ 130 mg/dL and Low HDL: HDL < 40 mg/dL in men and < 50 in women; (National Cholesterol Education Program, ATP III, 2001)

**Table 4:** Dyslipidemia prevalence based on isolated and mixed phenotypes

	Phenotypes	Total (%)	Male (%)	Female (%)	P- value
Academic staff	Isolated hypertriglyceridemia	35.5	31.6	45.2	0.027
	Isolated hypercholesterolemia	8.9	10.1	6.0	0.256
	Isolated low HDL	29.6	30.9	26.2	0.423
	Mixed dyslipidemia	14.2	14.0	14.3	0.951
Students	Isolated hypertriglyceridemia	24.9	28.9	18.2	0.085
	Isolated hypercholesterolemia	11.3	10.2	13.2	0.525
	Isolated low HDL	29.3	27.3	32.5	0.435
	Mixed dyslipidemia	14.1	19.5	5.2	0.004

P-values are obtained from the chi-square test. Isolated hypertriglyceridemia (Isolated hyperTG): TG ≥ 150 mg/dL and TC < 200 mg/dL; isolated hypercholesterolemia (Isolated hyperTC): TC ≥ 200 mg/dL and TG < 150 mg/dL; isolated low HDL-C: HDL-C ≤ 40 mg/dL in men and ≤ 50 mg/dL in women without hypertriglyceridemia or hypercholesterolemia and Mixed hyperlipidemia: TG ≥ 150 mg/dL and TC ≥ 200 mg/dL (National Cholesterol Education Program, ATP III, 2001)

### Factors associated with dyslipidemia

The results of the multivariable logistic regression analysis for staff and students are presented in Table 5 and Table 6, respectively. Among staff members, hypertriglyceridemia was positively and independently associated with the 36–45 years age group. Hypercholesterolemia showed a positive association with 46–55 years and > 55 years age groups and inadequate physical activity. Elevated LDL showed a positive association with 36–45 years and 46–55 years and > 55 years age groups, and inadequate physical activity. Low HDL showed a positive association with general obesity and inadequate physical activity. In contrast, among students, hypertriglyceridemia showed a positive association with > 21 years of age groups and diabetes. Hypercholesterolemia was positively associated with abdominal obesity. Elevated LDL was positively associated with abdominal obesity and inadequate physical activity. Low HDL showed a positive association with > 21 years of age groups, diabetes, and inadequate physical activity.

**Table 5:** Assessing factors associated with dyslipidemia among academic staff

Variables	Elevated TG		P-value	Elevated TC	P-value	Elevated LDL	P-value	Low HDL	P-value
	OR (95% CI)			OR (95% CI)		OR (95% CI)		OR (95% CI)	
Age (years)									
18–35	Reference			Reference		Reference		Reference	
36–45	0.38 (0.16–1.46)		0.046	3.38 (0.64–7.67)	0.149	3.49 (0.90-13.48)	0.070	0.93 (0.48–1.82)	0.832
46–55	1.68 (0.45–6.24)		0.433	6.63 (1.21–8.34)	0.029	4.18 (0.98–17.74)	0.045	1.91 (0.73–4.96)	0.184
> 55	1.86 (0.27–6.70)		0.798	7.91 (0.45–9.70)	0.042	5.77 (1.23–19.65)	0.048	1.55 (0.40–6.08)	0.528
Gender									
Male	Reference			Reference		Reference		Reference	
Female	0.52 (0.23–1.19)		0.120	1.11 (0.05–13.43)	0.945	0.55 (0.04-8.00)	0.661	0.76 (0.30–1.93)	0.569
BMI (kg/m^2^)									
Normal	Reference			Reference		Reference		Reference	
Overweight	1.77 (0.58–5.42)		0.316	1.78 (0.17–20.33)	0.998	6.89 (0.07–17.23)	0.998	1.58 (0.53–4.70)	0.409
Obese	1.60 (0.40–6.32)		0.503	1.53 (0.07–17.16)	0.998	7.5 (0.05–21.87)	0.998	8.88 (1.83–23.10)	0.007
Diabetes									
No	Reference			Reference		Reference		Reference	
Yes	0.73 (0.31–1.72)		0.471	0.93 (0.17–4.95)	0.930	1.31 (0.31–5.52)	0.706	1.51 (0.63–3.65)	0.353
Smoking									
No	Reference			Reference		Reference		Reference	
Yes	0.77 (0.12–4.95)		0.779	1.44 (0.03–13.56)	0.996	1.48 (0.01–8.78)	0.867	0.56 (0.03–9.58)	0.689
Physical activity									
Medium/Adequate	Reference			Reference		Reference		Reference	
Inadequate	0.73 (0.32–3.62)		0.435	0.76 (0.16–2.56)	0.032	0.75 (0.21–2.70)	0.036	2.53 (1.01–6.38)	0.049

Values are presented as OR (95% CI). OR = Odds ratio, CI = Confidence Interval. Multivariable logistic regression was applied to evaluate the relationship between elevated lipid profile markers and associated factors

**Table 6:** **A**ssessing factors associated with dyslipidemia among students

Variables	Elevated TG	P-value	Elevated TC	P-value	Elevated LDL	P-value	Low HDL	P-value
	OR (95% CI)		OR (95% CI)		OR (95% CI)		OR (95% CI)	
Age (years)								
18–21	Reference		Reference		Reference		Reference	
22–24	4.38 (1.92–9.91)	0.000	7.46 (0.77–5.45)	0.089	1.59 (0.47–5.32)	0.451	5.75 (2.39–13.84)	0.000
> 24	3.59 (1.34–9.54)	0.010	7.75 (0.67–9.17)	0.104	0.91 (0.19–4.38)	0.913	4.44 (1.56–12.68)	0.005
Gender								
Male	Reference		Reference		Reference		Reference	
Female	0.34 (0.07–1.73)	0.195	0.57 (0.02–17.94)	0.748	0.60 (0.02–16.58)	0.762	0.45 (0.09–2.34)	0.341
BMI (kg/m^2^)								
Normal	Reference		Reference		Reference		Reference	
Overweight	2.97 (0.80–11.00)	0.103	0.67 (0.02–12.82)	0.821	3.39 (0.01–11.62)	0.998	1.48 (0.27–8.22)	0.656
Obese	1.46 (0.86–15.10)	0.065	1.79 (0.05–9.14)	0.998	1.02 (0.01–1.32)	0.998	0.56 (0.03–9.08)	0.680
Diabetes								
No	Reference		Reference		Reference		Reference	
Yes	1.86 (1.31–2.62)	0.000	1.46 (0.78–2.72)	0.234	1.88 (1.14–3.11)	0.013	1.57 (1.19–2.04)	0.001
Smoking								
No	Reference		Reference		Reference		Reference	
Yes	8.29 (0.56–12.67)	0.122	1.46 (0.03–14.80)	0.999	1.18 (0.03–10.38)	0.998	0.79 (0.05–12.26)	0.867
Physical activity								
Medium/Adequate	Reference		Reference		Reference		Reference	
Inadequate	0.44 (0.07–2.75)	0.380	5.18 (0.26–10.56)	0.278	3.39 (1.22–5.71)	0.032	3.30 (0.31–4.84)	0.032

Values are presented as OR (95% CI). OR = Odds ratio, CI = Confidence Interval. Multivariable logistic regression was applied to evaluate the relationship between elevated lipid profile markers and associated factors

## Discussion

The prevalence of dyslipidemia is steadily increasing over the past few decades and has become a global public health problem. Its prevalence varies widely according to ethnicity, socioeconomic status, culture, lifestyle, and dietary habits. This study determined the prevalence and associated risk factors among university academic staff members and students in Bangladesh. In this study, the prevalence of dyslipidemia was 85% in academic staff and 76.5% in students. In both academic staff and students, the most prevalent form of dyslipidemia was low HDL-C.

Some previous studies also determined the prevalence of dyslipidemia among staff and students in Asian countries. For example, a recent study conducted by Zhou et al. reported the prevalence of dyslipidemia among university staff members in China [32]. The authors reported comparatively an increased prevalence of dyslipidemia in male (51.49%) than in female (41.77%) staff members, with no significant difference between the genders [32]. In the present study, the prevalence of dyslipidemia was higher than those reported by Zhou et al. [32]. Similar to that study findings, we also did not find a significant difference in the prevalence of dyslipidemia between male (83.5%) and female staff (88.7%) members. This might be a reason the study participant’s education levels were high. It has been suggested that both lifestyle and biological factors are associated with dyslipidemia, which can be changed by education level; and a significant impact of higher education level was observed on TC and LDL-C components [33]. However, the exact mechanism by which the education level has an impact on dyslipidemia is not well understood, they likely involve psychological stress, unhealthy dietary habits and an unbalanced lifestyle [32]. A recent study showed that about 89% of university staff had moderate/high stress and only 25% of staff slept at least 8 h nightly [34]. There is also evidence that stress can increase the risk of obesity, diabetes, hypertension, and CVD [35]. Similar findings were found in other studies, showed that workers in pressurized environments or previous work stress history were possibly to have CVD [36, 37]. There are some possible mechanisms by which work-related stress can influence the pathways of cardiovascular pathology at molecular level, including high secretion of inflammatory cytokines and cortisol [35, 38, 39]. On the other hand, some studies have demonstrated a higher prevalence of dyslipidemia and a lower rate of awareness and treatment among males than among females [40, 41]. There are many disciplines in a university; however, health-related education is covered in a few disciplines, therefore, health education and intervention programs for university staff members are needed to reduce and prevent dyslipidemia.

The prevalence of dyslipidemia in our student cohort is close to the prevalence rate found among students (86.7%) of a Yemeni University [10]. Comparatively, a lower prevalence of dyslipidemia was found among university students (60%) in Saudi Arabia [9]. Another study also reported a low prevalence of dyslipidemia among students (63.8%) in a university in Egypt [42]. In our study, the prevalence of lipid profile abnormalities is comparatively higher (except high LDL) than those reported in a recent study conducted on undergraduate Medical College students (n = 100) in Dhaka, where the prevalence of hypertriglyceridemia, hypercholesterolemia, high level of LDL and low level of HDL were: 28.0%, 22.0%, 30.0%, 31.0%, and respectively [43]. This lower prevalence in that study [43] might be related to the small number of participants enrolled from a single institution or some differences in food habits. In the present study, male students had a comparatively higher prevalence of dyslipidemia than females, although the prevalence difference was not statistically significant between the genders. A study in Pakistan showed an increased tendency of dyslipidemia in younger males compared to younger females [44]. In other studies, an insignificant difference was also found in dyslipidemia prevalence between the genders [45, 46]. This variation between the gender groups in young adults might be related to food consumption habits and/or some physiological differences. In the present study, we also observed a high prevalence of dyslipidemia in female staff than the female students. This higher level of dyslipidemia among female staff might be related to increased age and postmenopausal effects on lipid levels. Epidemiological studies suggested that menopause may have a potential role in altering TC, LDL-C and HDL-C levels [47–49].

In this study, the prevalence of high TC, high LDL, and low HDL in the 36–55 years age group in the staff member is a concern. However, the prevalence of hypertriglyceridemia was higher in the 18–35 years age group, although it differs within the age groups. In our study subjects, the factor that contributed to hypertriglyceridemia might be a carbohydrate-rich diet [50]. In our study, hypercholesterolemia and elevated LDL were significantly associated with increased age. The possible reason might be the excess deposition of visceral fat which leads to releasing of high levels of free fatty acids and pro-inflammatory cytokines from the adipocytes and related macrophages, which further influence insulin resistance with increased age [51–53]. A retrospective study that included a large number of participants showed a peak prevalence of dyslipidemia in 40–59 years of age in males and 60–69 years of age in females [54]. However, the exact mechanisms behind the differences in dyslipidemia risk in males and females are not clear yet.

It is well established that obesity is one of the vital contributors to developing dyslipidemia. In our study, both general and abdominal obesity were significantly associated with dyslipidemia. Similar results were found in previous studies in other countries [32, 42, 55, 56]. Increased weight gain among our participants may be related to a significant portion of sedentary behaviours and more deskbound activities. In epidemiological studies, it has been suggested that the association of abdominal obesity with dyslipidemia is mediated via an etiopathological mechanism [57]. So, increased BMI and WC are considered primary screening tools for detecting dyslipidemia individuals. Considering these aspects, controlling body weight and reducing body fat can be effective strategies to control dyslipidemia and hypertension.

Inadequate physical activities were related to dyslipidemia in our analysis. Similar findings were observed in several early studies [42, 46, 58]. Intervention studies indicated that less exercise as physical activity may elevate lipid profile marker levels, resulting in a decrease in TG concentrations and an increase in HDL-C concentrations [59, 60]. Thus, encouraging regular physical activities may be effective in controlling and reducing dyslipidemia. The most prevalent form of dyslipidemia was low HDL in our study, which was in line with other studies conducted in neighbouring countries. Whether low HDL-C contributed to the increased cardiovascular risk in the South Asian population remains unknown, more studies are required to evaluate this further.

The association of dyslipidemia with hypertension and diabetes has been reported in numerous studies [61–65]. Similar to these previous studies, we also found an increased prevalence of dyslipidemia among hypertensive and diabetic participants than among the healthy control participants. Dyslipidemia may affect arteries’ structure and function, impairs endothelial function, interrupt nitric oxide production and blood pressure regulation, and promotes atherosclerosis [63, 66]. It is well known that dyslipidemia is a significant risk factor for CVD. Increased blood glucose levels combined with dyslipidemia increase atherosclerosis-related inflammation and make it more complicated [67]. On the other hand, the accumulation of visceral fat leads to insulin resistance which may play a key role in inducing diabetic dyslipidemia [68].

Our study had also some limitations which need to consider. Firstly, due to the nature of the cross-sectional design, causality could not be established in our study. Secondly, our findings may not apply to other populations, as the study subjects were mainly university staff and students. Thirdly, we did not have detailed data on participants’ food habits. Finally, the sample size was relatively small; therefore, further large-scale studies are needed to determine the actual scenario of the prevalence and risk factors of dyslipidemia in these special population groups in Bangladesh. The major strength of the present study is that it provided important information on the increased prevalence of dyslipidemia and potential associated factors in university academic staff and students in Bangladesh. Furthermore, this study’s findings might be a foundation for further investigations to reduce the burden of dyslipidemia and related complications among these special groups of the national population.

## Conclusion

This study indicated a high prevalence of dyslipidemia among academic staff and students in Bangladesh. Low HDL-C was the most prevalent form of dyslipidemia among the study participants, followed by higher TG. The risk of dyslipidemia was significantly related to increased age, obesity, diabetes and inadequate physical activity. These results suggest the need for a screening program for the study of blood lipids and proper intervention programs to reduce and prevent dyslipidemia among academic staff and students in Bangladesh.

## Data Availability

The datasets analyzed in the current study are available from the corresponding author upon reasonable request.
